# Biotech *Eucalyptus* can sustainably address society’s need for wood: the example of freeze tolerant *Eucalyptus* in the southeastern U.S

**DOI:** 10.1186/1753-6561-5-S7-I24

**Published:** 2011-09-13

**Authors:** Maud Hinchee, Chunsheng Zhang, Shujun Chang, MIchael Cunningham, William Hammond, Narender Nehra

**Affiliations:** 1 Les Pearson ArborGen Inc., P.O. Box 840001, Summerville, South Carolina, USA

## Background

*Eucalyptus* species are among the fastest growing woody plants in the world and represent about 8% of all planted forests (~18 million hectares) grown in 90 countries [1]. Only a limited number of species are grown commercially, and these have been the focus of extensive breeding to improve desirable wood properties such as basic density, cellulose content, fiber length and improved growth. Improved *Eucalyptus* varieties grown in managed plantations as a source of timber and pulpwood have provided socio-economic benefits for both small and large land owners. *Eucalyptus* also provides a cost-effective source of lignocelluloses for the production of energy and advanced biofuels. However, the large scale plantings of the most productive *Eucalyptus* species in the Southeast U.S. are currently limited to regions of central and southern Florida where no hard freezes occur in winter. Plantings of hardwood species to support the traditional forest industry in the Southeastern U.S. are infrequent due to slow growth rates and high plantation establishment and management costs. The biotech Freeze Tolerant *Eucalyptus* would provide an economically viable plantation hardwood for the Southeastern U.S.

## Methods

Genetically engineered Freeze Tolerant Eucalyptus (AGEH427) was developed by the introduction of plasmid pABCTE01 into the EH1 genotype of *E. grandis x E. urophylla* using the *Agrobacterium* transformation system described by Cheah (2001) [2]. pABCTE01 contains a CBF2 expression cassette comprised of Arabidopsis cold-inducible promoter rd29A [3] driving the Arabidopsis *CBF2* (C-Repeat Binding Factor) cDNA [4,5]. It also contains a pollen control cassette consisting of a modified *barnase* gene from *Bacillus amyloliquefaciens* under the control of a *Pinus radiata* anther-specific promoter (PrMC2) [6]. Single insert, backbone free transgenic lines were vegetatively propagated to enable 21 replicated field trials established at eight different locations representing USDA Hardiness Zones 8a (potential kill zone), 8b (target freeze-stress zone), and 9a ( freeze stress-free zone) across the Southeast US. A simple comparison of pre-winter and post-winter live height measurements was used to calculate a percent dieback of the main stem, and provided appropriate assessment of freeze tolerance and growth performance under field conditions. Data were collected over five winter/growing seasons. Pollen ablation was studied by collecting multiple flowers from field grown transgenic and non-transgenic trees prior to anthesis and microscopic assessment of the frequency of normal pollen.

## Results

The transgenic line AGEH427, had significant freeze hardiness that enabled it to grow in USDA Hardiness Zone 8b. The growth of AGEH427 was comparable to EH1 non-transgenic control trees when grown under low- or no-freeze challenge conditions (Zone 9a). In the target freeze-challenged environment (Zone 8b), AGEH427 grew better than the non-transgenic control. After the severe winter of 2009/2010 at a site with a minimum temperature of 16.8 °F, the average live height of control trees was approximately 0.3 feet as compared to 52.4 feet for AGEH427. AGEH427 had approximately 10% dieback from the top. In the zone 8a (winter temperatures as low as 8.4 °F), complete dieback and defoliation were observed, with post-winter tree heights for both AGEH427 and the non-transgenic control being only 0.1 ft. AGEH427 also did not produce pollen, whether or not it was grown in freeze challenged locations. No pollen was produced over multiple years, different flowering seasons, different sites, and different physiological ages of trees while the control trees did produce pollen. Additional phenotypic evaluations of AGEH427 in comparison to the control were performed in field studies across a broad range of environmental conditions in the Southeast U.S., and no differences, other than the freeze tolerance and pollen ablation traits were observed. Neither the transgenic nor the control trees demonstrated any tendency to spread beyond a managed plantation, with low seed set and lack of any seeded volunteers observed in the field trials.

## Conclusions

The cumulative multi-season data obtained from these trials demonstrated conclusively that the freeze tolerant trait in AGEH427 enabled this tropical tree to grow in the subtropical, freeze-prone USDA Hardiness Zone 8b. The productivity of AGEH427 makes it ideal as a short-rotation plantation hardwood in the Southeast U. S. The yield achievable with freeze tolerant *Eucalyptus* is predicted to meet or exceed the required productivity for an economically feasible hardwood plantation to meet traditional forest industry and bioenergy needs [7]. There is no evidence from the literature or from our field trials which indicates that EH1-427 would be invasive or negatively impact endangered species. EH1-427 is currently the subject of a petition for deregulation in the U.S.

## References

[B1] FAO State of the World’s Forests Food and Agriculture Organization 2007

[B2] Cheah K Methods for producing genetically modified plants, plant materials and plant products produced thereby United States Patent 6,255,559 B1 2001

[B3] Yamaguchi-Shinozaki Shinozaki K Characterization of the expression of a desiccation-responsive *rd29* gene of *Arabidopsis thaliana* and analysis of its promoter in transgenic plants Mol Gen Genet 1993 236 331 340 10.1007/BF00277130 8437577

[B4] Jaglo-Ottosen K Gilmour S Zarka D Schabenberger O Thomashow M *Arabidopsis* CBF1 overexpression induces COR genes and enhances freezing tolerance Science 1998 280 104 106 10.1126/science.280.5360.104 9525853

[B5] Zhang X Fowler S Cheng H Lou Y Rhee S Stockinger E Thomashow M Freezing-sensitive tomato has a functional CBF cold response pathway, but a CBF regulon that differs from that of freezing-tolerant *Arabidopsis* Plant J 2004 39 905 919 10.1111/j.1365-313X.2004.02176.x 15341633

[B6] Rottmann W Norris-Caneda K Zhang C Reproductive ablation constructs United States Patent 7851679 2010

[B7] English B De La Torre Ugarte D Jensen K Hellwinckel C Menard J Wilson B Roberts R Walsh M 25% Renewable Energy for the United States by 2025: Agricultural and Economic Impacts 2006 University of Tennessee Agricultural Economics http://www.agpolicy.org/ppap/REPORT%2025x25.pdf

